# Polymyalgia Rheumatica Revealing a Lymphoma: A Two-Case Report

**DOI:** 10.1155/2016/2986297

**Published:** 2016-08-11

**Authors:** Frank Verhoeven, Xavier Guillot, Mickaël Chouk, Clément Prati, Daniel Wendling

**Affiliations:** Department of Rheumatology, CHRU Besançon, 2 Boulevard Fleming, 25030 Besançon, France

## Abstract

*Introduction*. Polymyalgia rheumatica (PMR) is one of the most common inflammatory rheumatism types in elderly population. The link between cancer and PMR is a matter of debate.* Methods*. We report two cases of PMR leading to the diagnosis of lymphoma and the growing interest of PET-TDM in this indication.* Results*. A 84-year-old man known for idiopathic neutropenia presented an inflammatory arthromyalgia of the limb girdle since one month. Blood exams highlighted the presence of a monoclonal B cell clone. Bone marrow concluded to a B cell lymphoma of the marginal zone. He was successfully treated with 0.3 mg/kg/d of prednisone, and response was sustained after 6 months. A 73-year-old man known for prostatic neoplasia in remission for 5 years presented arthromyalgia of the limb girdle since one month. PET-CT revealed bursitis of the hips and the shoulders, no prostatic cancer recurrence, and a metabolically active iliac lymphadenopathy whose pathologic exam concluded to a low grade follicular lymphoma. He was successfully treated with 0.3 mg/kg/d of prednisone.* Conclusion*. These observations may imply that lymphoma is sometimes already present when PMR is diagnosed and PET-CT is a useful tool in the initial assessment of PMR to avoid missing neoplasia.

## 1. Introduction

Polymyalgia rheumatica (PMR) is one of the most common inflammatory disorders in elderly population [1]. Its diagnosis is often difficult and consists in an addition of clinical, biological, and ultrasonographic features [2]. The pathogenesis of PMR is not well established and may appear as an isolated syndrome or occur concomitantly with giant cell arteritis or cancer, in a setting of a paraneoplastic syndrome [3]. The link between cancer and PMR is a matter of debate [4]. This paraneoplastic syndrome is more common with solid tumors, whereas hematological malignancies associated cases are rare. Lymphoma is a malignant pathology of the immune system. It could be a consequence of, or associated with, autoimmune and inflammatory diseases [5]. This link between lymphoma and PMR is known and it shows the implication of B lymphocyte in the physiopathology of these autoimmune diseases [6, 7].

## 2. Cases Presentation

### 2.1. Case 1

A 84-year-old man, with a history of idiopathic neutropenia, presents with an inflammatory arthromyalgia of the limb girdle for one month. He described a morning stiffness over an hour and an improvement of the symptoms during the day. There were neither synovitis nor arguments for giant cell arteritis. The X-ray imaging was normal and the blood exams highlighted an inflammatory syndrome (CRP: 53 mg/l), a neutropenia (1200 G/mm^3^), no autoantibodies, and the presence of a monoclonal B cell clone. Bone marrow aspiration revealed lymphocytic infiltration lower than 1% and concluded to a B cell lymphoma of the marginal zone. The thoracoabdominal CT scan showed multiple mediastinal and cervical lymphadenopathies. No treatment was necessary for the lymphoma and the hematologists gave their agreement for a treatment with prednisone for the management of the PMR. Symptoms were controlled after 3 days of treatment with 0.3 mg/kg/d of prednisone with a sustained response after 12 months of treatment with decreasing dose of prednisone.

### 2.2. Case 2

**Figure 1:**
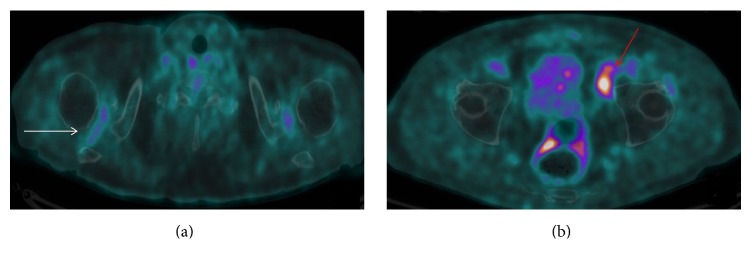
PET-CT pictures of symmetrical shoulders hypermetabolic bursitis (a) and of metabolic active iliac lymphadenopathy (b).

A 73-year-old man, with a history of prostatic neoplasia treated by prostatectomy and in remission for 5 years, presented with arthromyalgia of the limb girdle for one month. The blood examinations revealed an inflammatory syndrome (CRP 63 mg/l), undetectable PSA, and no autoantibodies (rheumatoid factor and antinuclear and anti-CCP negative). Finally, a PET-CT revealed a metabolically active iliac lymphadenopathy, no prostatic cancer recurrence, and bursitis of the hips and the shoulders (Figure 1). The pathologic exam of this lymphadenopathy concluded to a low grade follicular lymphoma. No treatment was necessary for the lymphoma and the hematologists gave their agreement for the prednisone therapy. The patient was successfully treated with 0.3 mg/kg/d of prednisone with a disappearance of the inflammatory symptoms after 3 days of treatment. Six months later, the inflammatory symptoms were still controlled by prednisone but lymphoma progressed, needing rituximab.

## 3. Discussion

These cases suggest a possible link between PMR and lymphoma. This association has been described in the initial stage of the symptoms and only three other cases are described in the PubMed database [8–10]. At the same time, it is established that there is a risk of lymphoma in case of PMR [6, 7]. In these studies, the standardized incidence ratios for non-Hodgkin's lymphoma and Hodgkin's lymphoma were, respectively, 1.4 (95% IC: 1.2–1.6) and 2.2 (95% IC: 1.4–3.5). Interestingly, these lymphomas were mostly diagnosed in the first year following the diagnosis of polymyalgia rheumatica. These observations may imply that lymphoma is sometimes already present when PMR is diagnosed and is initially improved with prednisone.

In the second case, the PET-CT demonstrated two advantages. First, it enabled the positive diagnosis of PMR and highlighted metabolic active lymphadenopathies. One other case of concomitant lymphoma to PMR was diagnosed using PET-CT. There is currently a growing interest for this imaging procedure [11]. Indeed, PET-CT establishes the positive diagnosis of PMR highlighting multiple inflammatory bursitis [12] in different anatomic sites (shoulder, hips, interspinous space, ischial tuberosity, and great trochanter). In addition, it helps in highlighting neoplastic processes that might go unnoticed in the initial stage of PMR. PET-CT could play an important role in the initial assessment of PMR to avoid missing neoplasia. Given the high cost of applying PET-CT, it should remain restricted to patients with neoplastic history and those not responding to prednisone.

In the future, it would be interesting to know if, with the contribution of PET-CT, the prevalence of PMR presenting as a paraneoplastic syndrome will increase and if the prevalence of lymphoma associated with PMR will increase.
